# Death of Dieffenbach

**Published:** 1848-02

**Authors:** 


### DEATH OF DIEFFENBACH.

This eminent surgeon has also paid the debt of nature. He died suddenly at Berlin in December last.

